# Supplementary material for: Simulators and Simulations for Extracorporeal Membrane Oxygenation: An ECMO Scoping Review
Source: J Clin Med. 2023 Feb 22;12(5):1765. doi: 10.3390/jcm12051765 (PMC10003420; doi:10.3390/jcm12051765)
Supplement: Supplementary file 1 [file jcm-12-01765-s001.zip › Figures S1 Graphical Summary of ECMO sims.pdf]

S1: Graphical Summary of ECMO sims

An overview of all the ECMO simulations and simulators (ECMO sims) used in the manuscript. The ECMO sims are in order of overall fidelity classification. For low-fidelity ECMO sims, see Figure S1-10. For mid-fidelity ECMO sims, see Figure S11-26.

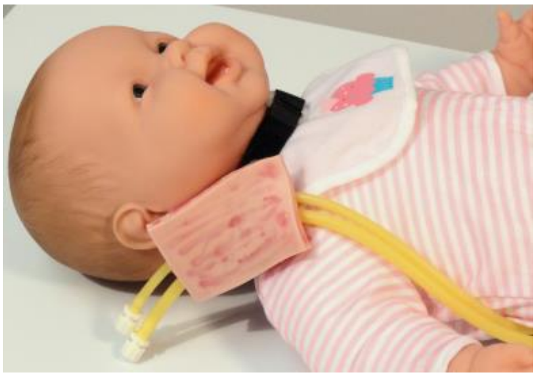

Figure S1. ECMO Simulation Kit by 3-Dmed® [1] is a low-fidelity ECMO sim, containing a cannulation pad and fluid-filled tubing mimicking vessels for cannulation.

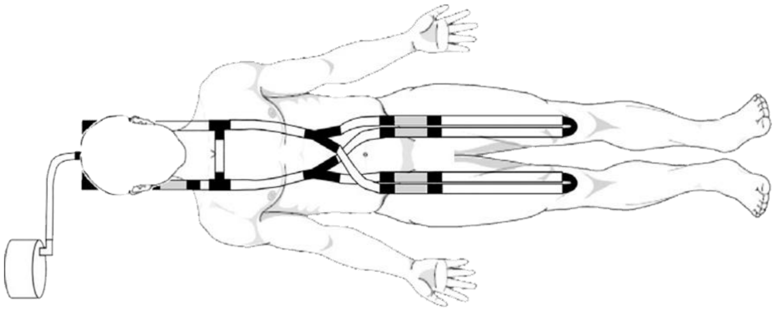

Figure S2. Endo Circuit by Endo *et al.* [2] is a low-fidelity ECMO sim, consisting a water tank, external fluid-filled polyvinyl chloride tubing, mimicking vessels for cannulation at multiple cannulation sites consisting of silicon tubes (grey parts), and joints (black parts).

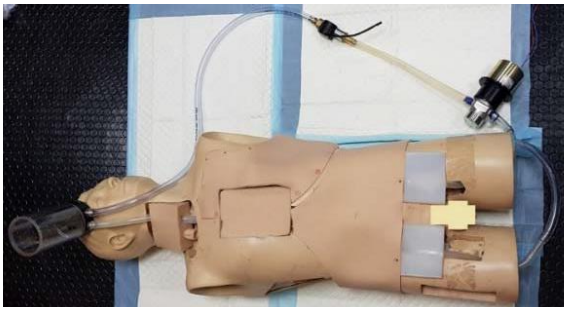

Figure S3. Cannulation Simulator by Mahmoud *et al.* [3] is a low-fidelity ECMO sim, consisting of an adult mannikin with implemented system and multiple integrated cannulation sites and fluid-filled mimicking the vessels for cannulation.

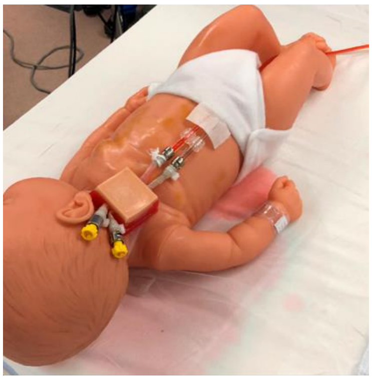

Figure S4. Surgical model by Palmer *et al.* [4] is a low-fidelity ECMO sim, consisting of three-layers silicon polymer mimicking skin tissue. The skin model contains liquid-filled tubing mimicking the vessels for cannulation. The skin model can be affixed on a neonatal model for cannulation practice.

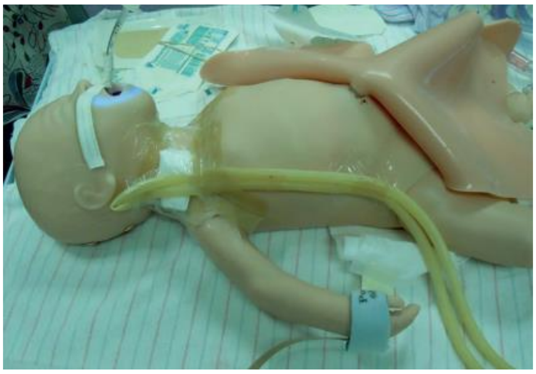

Figure S5. ECMO Initiation Simulator by Thompson *et al.* [5] is a low-fidelity ECMO sim, consisting of a neonatal mannikin with (partly) external cannulation sites and liquid-filled tubing mimicking the vessels for cannulation. Some parts of the tubing have been hidden by a cover (see femoral).

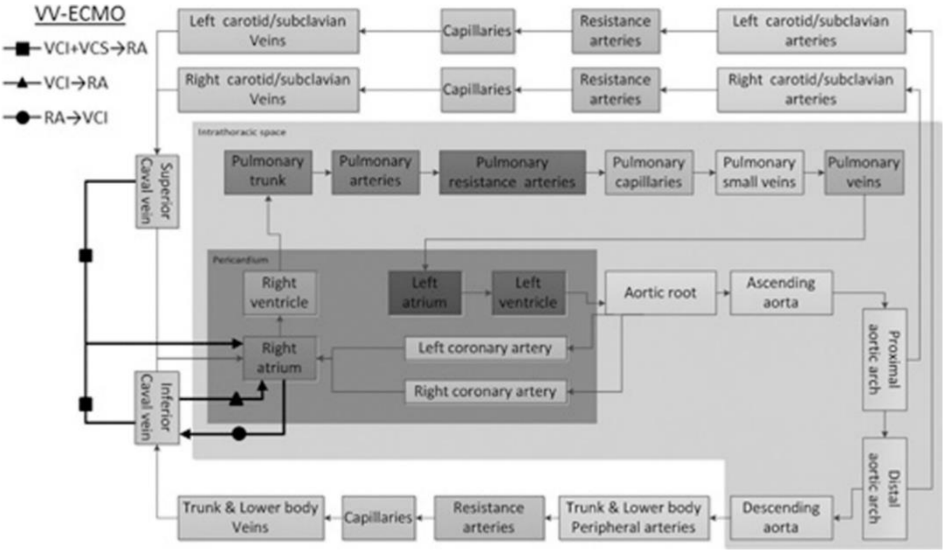

Figure S6. Aplysia by Broman *et al.* [6] is a low-fidelity ECMO sim with a closed-loop, real-time cardiovascular simulation model. A schematic representation of the cardiovascular components can be seen, consisting of pericardium (dark grey) and intra-thoracic space (light grey). Three different veno-venous cannulation modes are show with thick black lines. The model consists of 27 vascular segments, 4 cardiac chambers with corresponding valves, pericardium, and intrathoracic pressure.

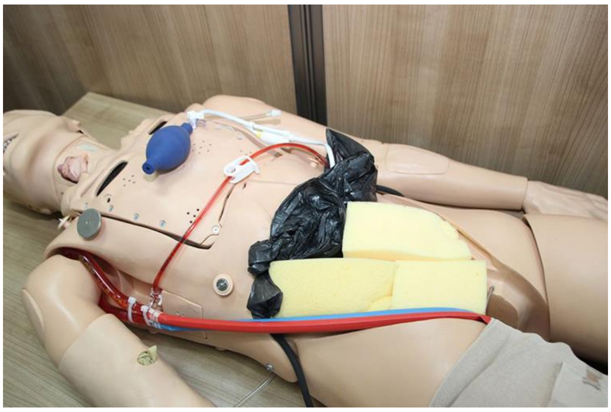

Figure S7. ECMO Therapy Simulator by Puślecki *et al.* [7] is a low-fidelity ECMO sim , using a commercially available full-body mannikin with an integrated electronic control unit, synthetic valves, pressure sensors, and hydraulic pumps.

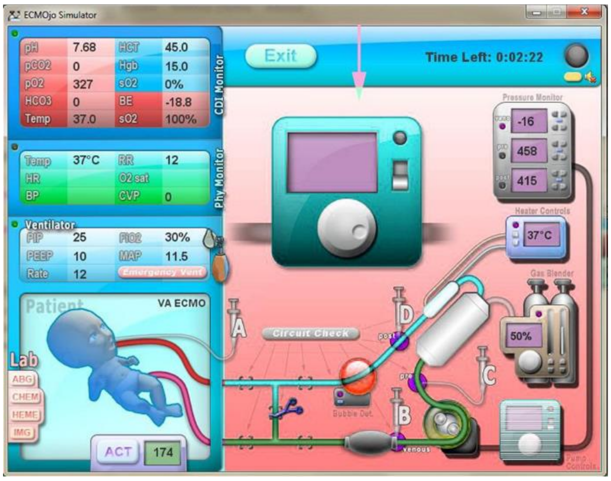

Figure S8. ECMOjo by Telehealth Research Institute [8] is a low-fidelity ECMO sim. It is an open-source computer application with several simulation options. The patient, circuit set-up, and the accompanying parameters are shown on screen.

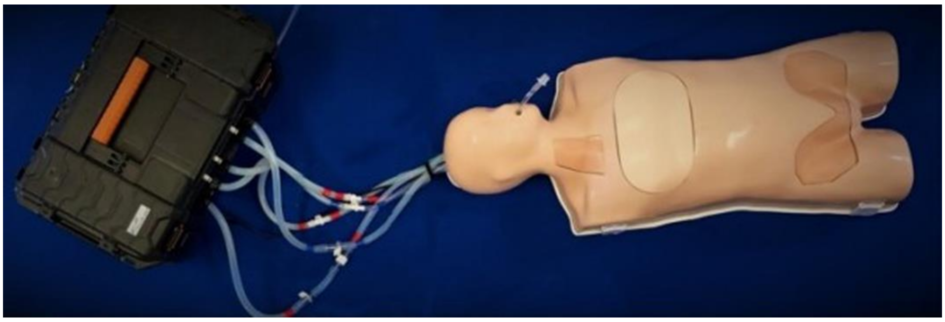

Figure S9. ECMO Trainer Evolution III by Creaplast [9] is a low-fidelity ECMO sim . This computational and physical model contains multiple cannulation sites and a cardiac massage simulator. A veno-arterial circulation passed through the liquid-filled tubing mimicking pulsatile vessels.

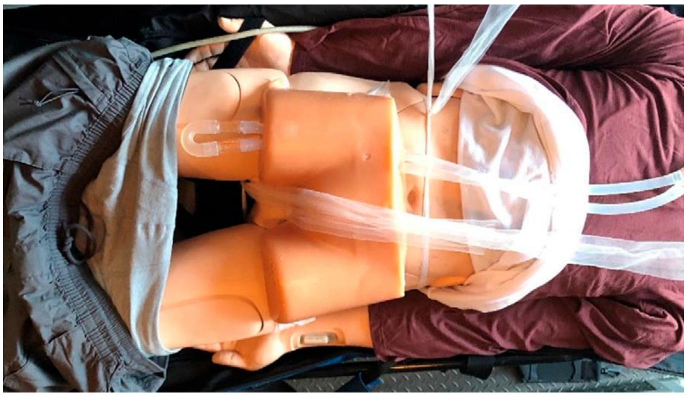

Figure S10. Percutaneous model by Palmer *et al.* [10] is a low-fidelity ECMO sim. It is a vascular ballistic gelatin model with fluid-filled tubing mimicking vessels for cannulation. The model can be affixed on mannikins for cannulation practice.

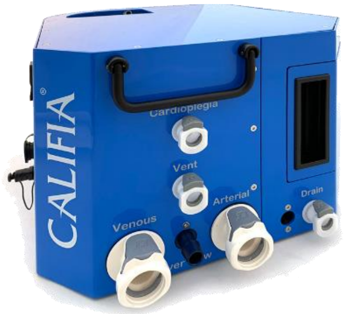

Figure S11. Califia Patient Simulator by BioMed Simulations [11] is a mid-fidelity ECMO sim. This device can be used for both ECMO and heart-lung machine simulations. It mimics the patient's hemodynamics, physiologic response, and respiratory changes.

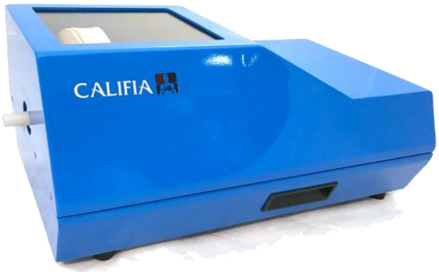

Figure S12. Califia Lung Simulator by BioMed Simulations [12] is a mid-fidelity ECMO sim. This device can be used for both ECMO and heart-lung machine simulations. It mimics the patient's hemodynamics, physiologic response (with interactive patient), respiratory changes, and simulates the patient's lung.

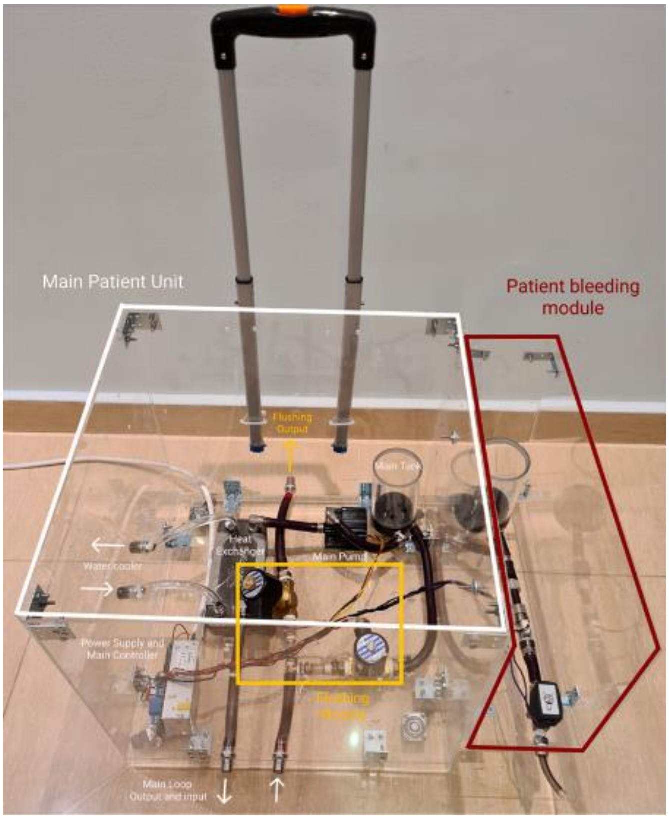

Figure S13. Modular ECMO Simulator by Alhomsi *et al.* [13] is a mid-fidelity ECMO sim. This device's casing cotains two modules (main patient unit and patient bleeding module) with liquid-filled tubing mimicking the vessels. Within the vessels oxygenation of blood is simulated using a thermochromic loop with a heater and cooling unit for change in blood color (light red and dark red). The device is modular and can therefore be extended with several components.

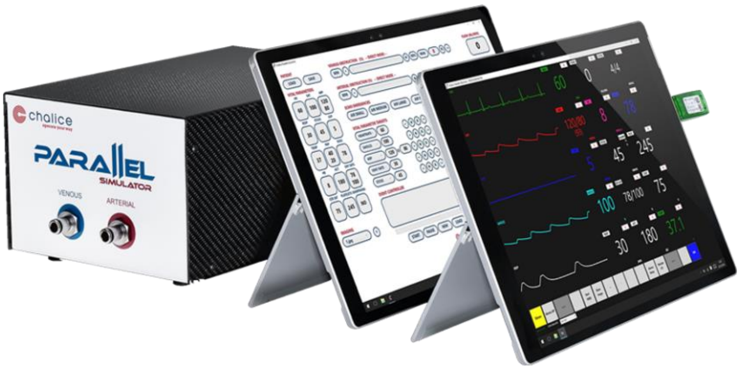

Figure S14. Parallel Simulator by Chalice [14] is a mid-fidelity ECMO sim. It is a portable and customizable simulation hub with two high-resolution tables (control panel and monitor). The Control panel sets vital statistics and physiological parameters can be altered to simulate situations. The monitor can be used by the trainee to interpret vital signs.

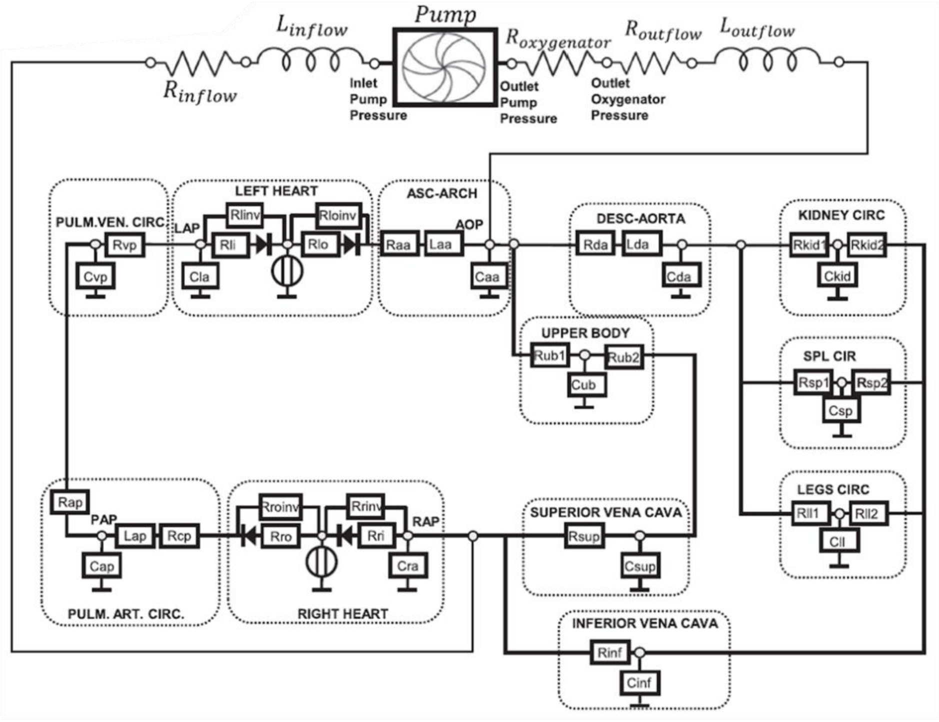

Figure S15. Computational ECMO Simulator by Colasanti *et al.* [15] is a mid-fidelity ECMO sim. A schematic overview of the electrical circuit of the main ECMO model components: resistances and inertances for inflow and outflow tubes, ECMO pump, and additional oxygenator resistance. The ECMO model is connected to the cardiovascular system including: left and right ventricles, atria, heart valves, and pulmonary and systemic circulations. The numerical model is validated and includes an hydraulic part, blood pump, oxygenator model, interface (main ECMO machine and Cardiac monitoring module).

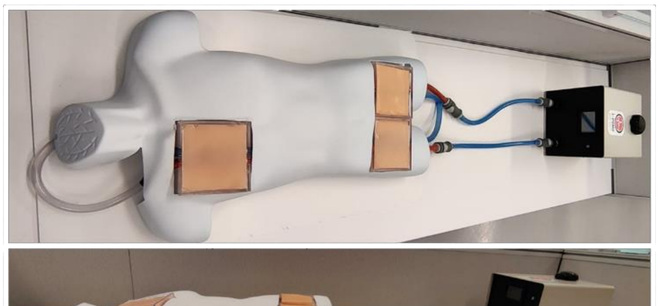

Figure S16. ECMO Training Simulator by Health Care Engineering Systems Center [16] is a mid-fidelity ECMO sim containing physiology software with pulsatile flow, hemodynamic simulations, and a blood substitute for change in blood color.

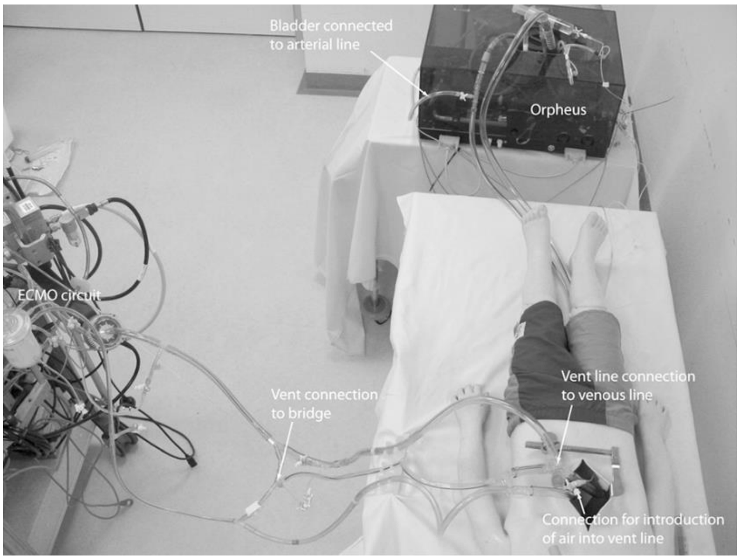

Figure S17. Orpheus Perfusion Simulator by Lansdowne *et al.* [17] is a mid-fidelity ECMO sim. A heart-lung machine (Orpheus perfusion simulator) is modified to an ECMO simulation model incorporating a resuscitation mannikin and oxygenation system

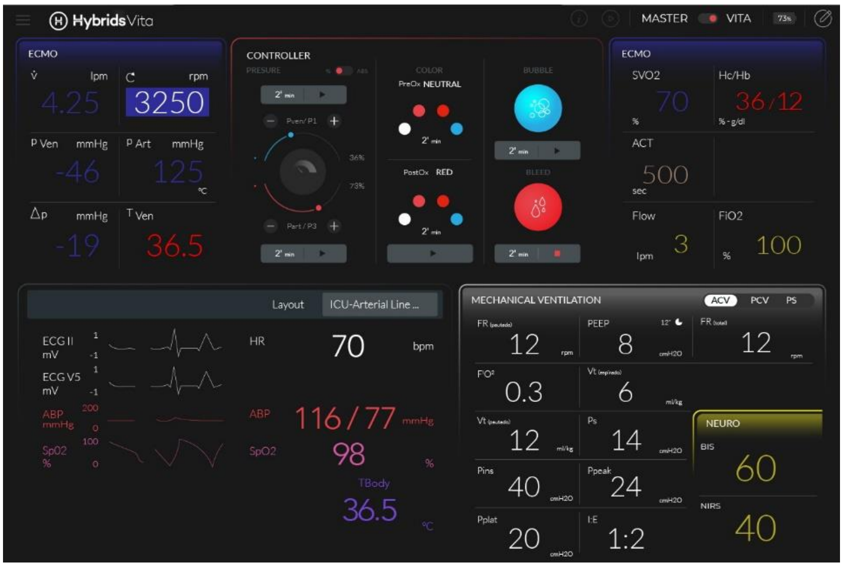

Figure S18. Hybrids Vita by Medical Simulator [18] is a mid-fidelity ECMO sim. This portable and wireless device captures the actual values of the connected ECMO machine and is integrated with: patient simulation, debriefing systems, mechanical ventilation, and brain monitoring software.

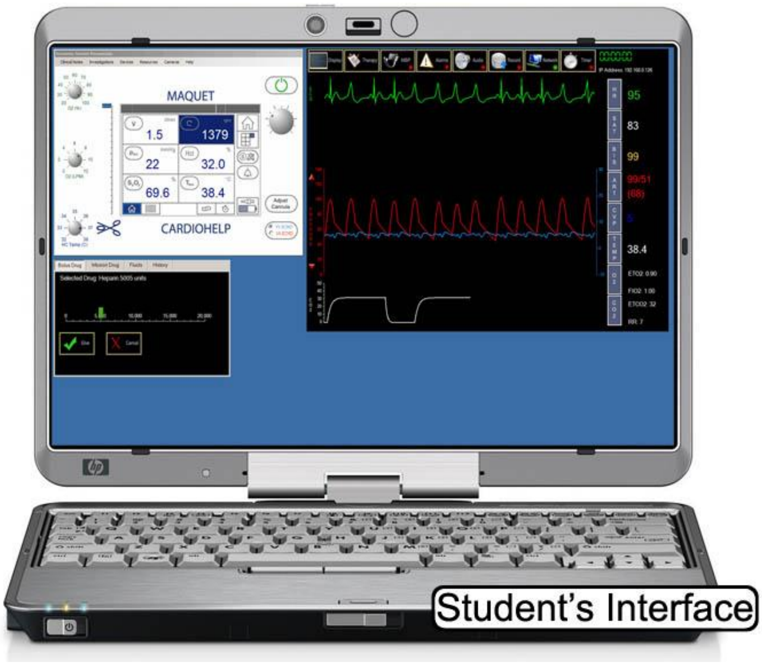

Figure S19. Adult ECMO Simulator by MSE [19] is a mid-fidelity ECMO sim. This screen-based computer application can be used for single use or small group tutorials. A separate application can remotely-control the system containing: a virtual' patient, centrifugal pump-based ECMO system, oxygenator, physiological monitor, ventilator, defibrillator, data recording system, and drugs and fluids for treatment.

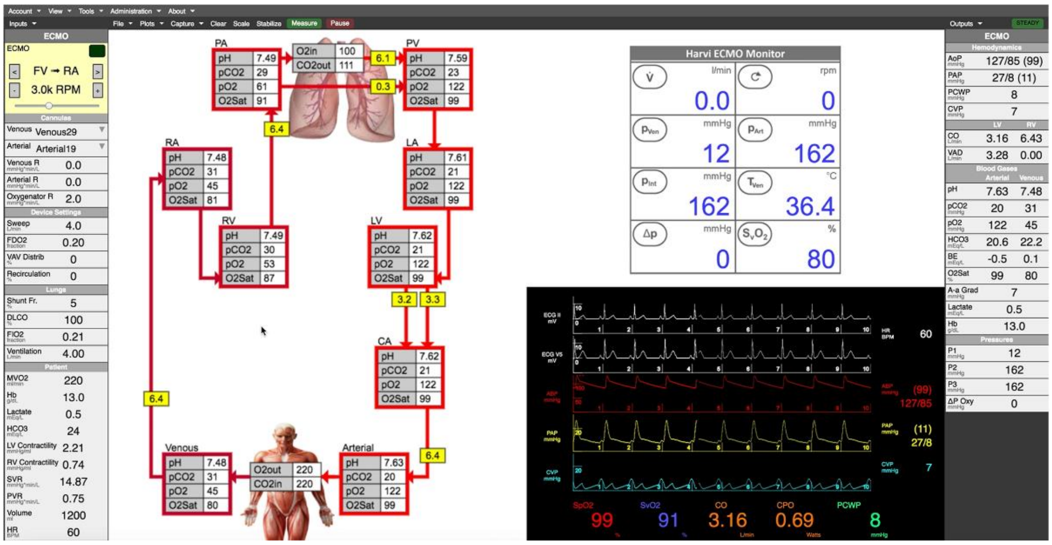

Figure S20. Harvi ECMO by PVLoops [20] is a mid-fidelity ECMO sim. This is an online training platform with guided tutorials. The interactive simulation contains several cannulation configurations and shows ECMO device parameters as well as patient output.

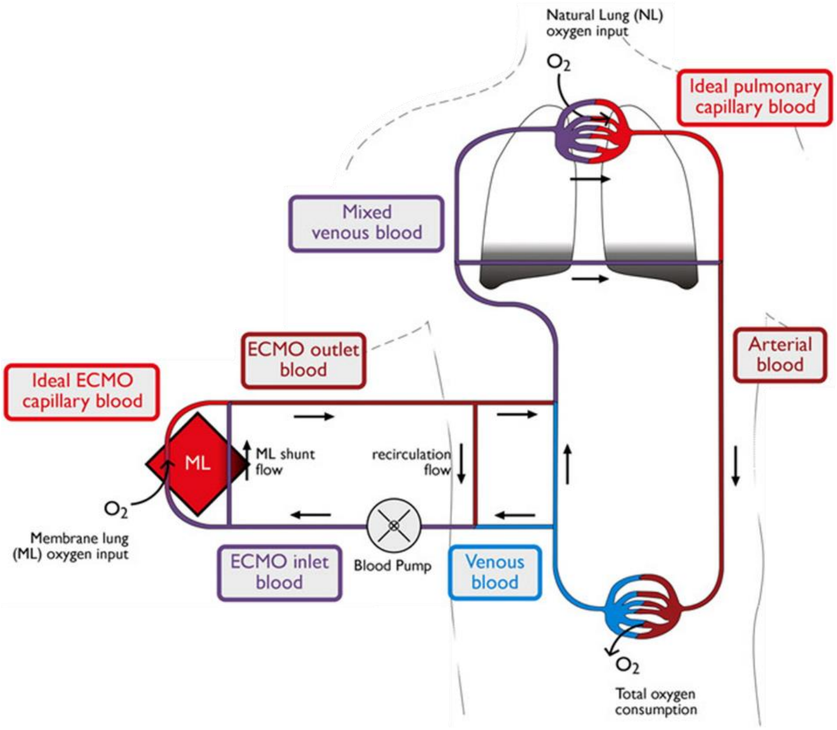

Figure S21. Mathematical ECMO model by Zanella *et al.* [21] is a mid-fidelity ECMO sim. This schematic representation of the numerical model consists of a simplified circulatory system of a patient connected to a veno-venous ECMO support. It contains seven different blood districts (arterial [Ca], venous, ECMO inlet, ECMO outlet, mixed venous, pulmonary capillary, and ML capillary), seven corresponding oxygen contents (Ca, venous, ECMO inlet, ECMO outlet, mixed venous, ideal pulmonary capillary, and ideal ML capillary), and numerical 2D and 3D graphical outputs.

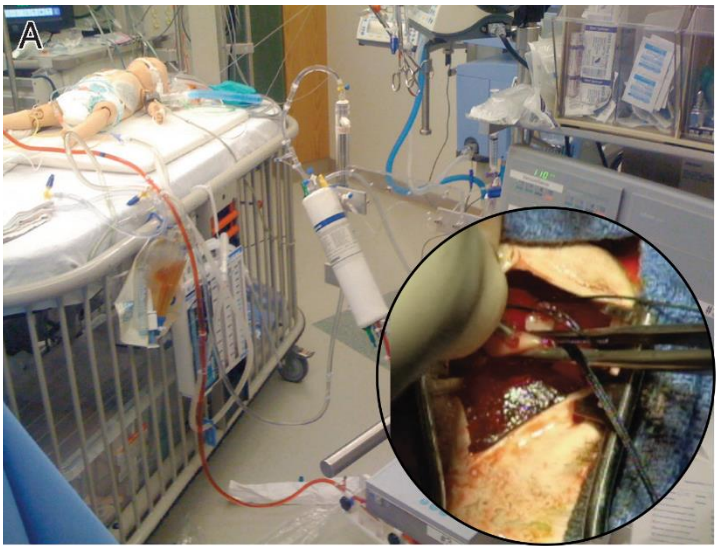

Figure S22. Integrated Skills Trainer by Allan *et al.* [22], is a mid-fidelity ECMO sim consisting of a cannulation inlay pad with blood-like fluid filling the silicon tubing representing the vessels.

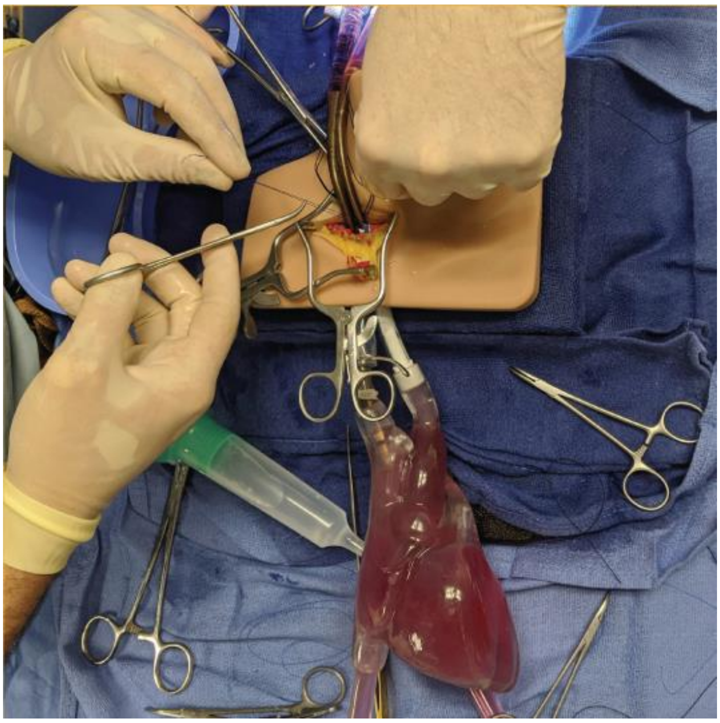

Figure S23. RediStik ECMO Cannulation Trainer by Texas Children's Hospital [23] is a mid-fidelity ECMO sim. A cannulation neck simulator with anatomical landmarks (mandible, clavicle, SCM), neurovascular positioning, color and feel, and possibility for exposure, cutting, and applying traction sutures/tourniquet controlling the vessels.

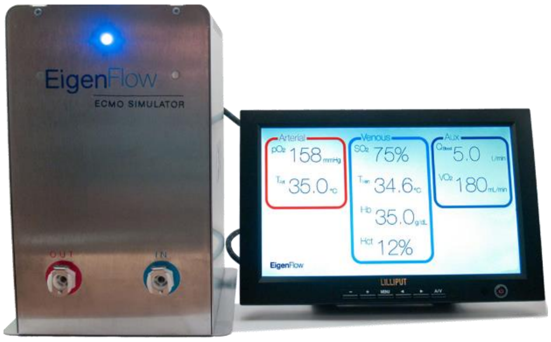

Figure S24. Eigenflow 2 ADVANCED by Curtis Life Research [24] is a mid-fidelity ECMO sim. A multifunction, wireless, and remote controlled simulator. The Eigenflow 2 ADVANCED can be connected to an ECMO circuit and has two variable controlled valved for clinical simulations. Furthermore, the 3-Dmed® ECMO cannulation kit [1] is used for cannulation training.

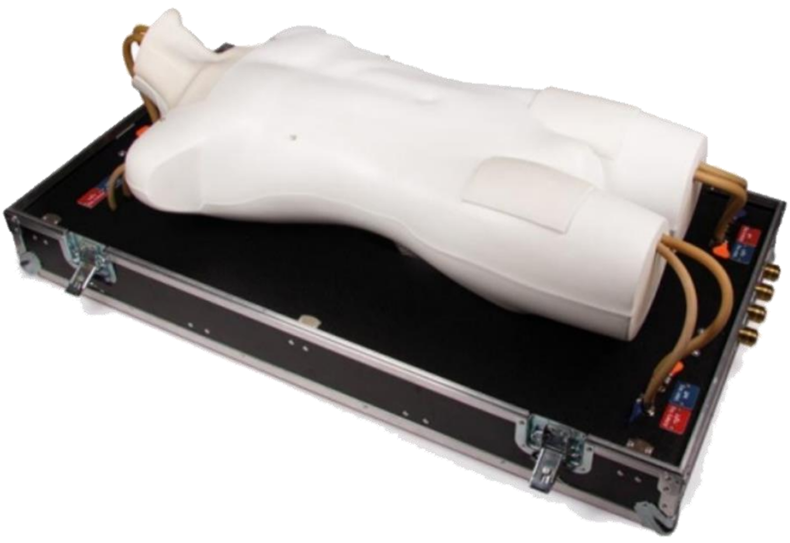

Figure S25. ECMO Trainer Professional MK2 by Erler Zimmer [25] is a mid-fidelity ECMO sim consisting of pump simulating arterial and venous circulation, an option for cannulation, and monitoring.

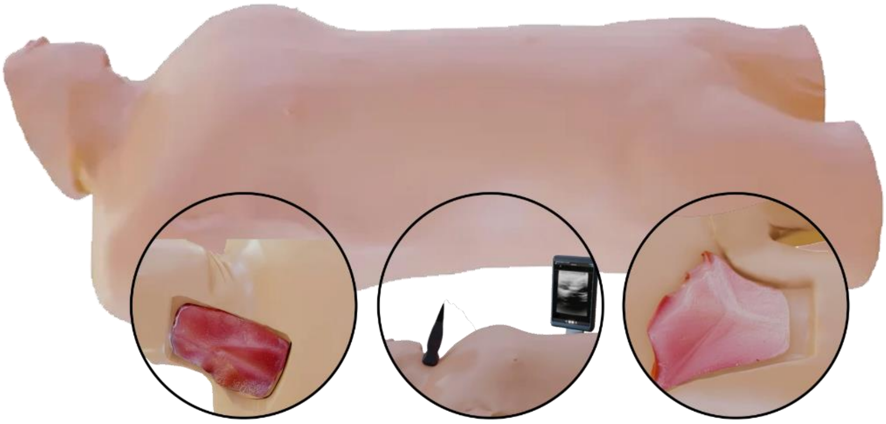

Figure S26. E-Sim Pro by The Simulator Company [26] is a mid-fidelity ECMO sim. Available options are: multiple cannulation site, connection to ECLS, heart rate control, cardiac output control, with physiological blood flow.

References

1. 3-Dmed. ECMO Simulation Kit. Available online: https://www.3-dmed.com/wp-content/uploads/2019/05/ECMO-Simulation-Kit.pdf (accessed on 10 October 2022).
2. Endo, T.; Kagaya, Y.; Arata, Y.; Imai, H. Long-term efficacy of an extracorporeal membrane oxygenation simulation with a novel, low-cost vascular model "Endo-Circuit". *Acute Medicine & Surgery* **2017**, *4*, 79-88, doi:10.1002/ams2.236.
3. Mahmoud, A.; Hssain, A.A.; Alinier, G.; Hassan, I.; Khurshid, U.; Abducarim, A.; Mahmud, S.; Abdallah, O.; Mohamed, E.; Alsalemi, A.; et al. Preliminary Implementation of the Next Generation Cannulation Simulator. 2019, 2019.
4. Palmer, D.; Aspenleiter, M.; da Silva, J.; Castro-Medina, M.; Morell, V.; Sharma, M.; Viegas, M. A High-Fidelity Surgical Model and Perfusion Simulator Used to Demonstrate ECMO Cannulation, Initiation, and Stabilization. *The Journal of ExtraCorporeal Technology* **2019**, *51*, 6.
5. Thompson, J.L.; Grisham, L.M.; Scott, J.; Mogan, C.; Prescher, H.; Biffar, D.; Jarred, J.; Meyer, R.J.; Hamilton, A.J. Construction of a reusable, high-fidelity model to enhance extracorporeal membrane oxygenation training through simulation. *Adv Neonatal Care* **2014**, *14*, 103-109, doi:10.1097/ANC.0000000000000054.
6. Broman, M.; Frenckner, B.; Bjällmark, A.; Broomé, M. Recirculation during Veno-Venous Extra-Corporeal Membrane Oxygenation – a Simulation Study. *The International Journal of Artificial Organs* **2015**, *38*, 23-30, doi:10.5301/ijao.5000373.
7. Puslecki, M.; Ligowski, M.; Kiel, M.; Dabrowski, M.; Stefaniak, S.; Sip, M.; Maciejewski, A.; Dabrowska, A.; Kiel-Puslecka, I.; Kłosiewicz, T.; et al. Prototype of extracorporeal membrane oxygenation (ECMO) therapy simulator used in regional ECMO program. *Journal of Thoracic Disease* **2018**, *10*, 5073-5079, doi:10.21037/jtd.2018.07.25.
8. ECMOjo Simulator. Available online: https://ecmojo.sourceforge.net/ (accessed on 12 October 2022).
9. ECMO Trainer Evolution III. Available online: https://www.crea-plast.com/en/45-ecmo-trainer (accessed on 12 October 2022).
10. Palmer, D.; Aspenleiter, M.; da Silva, J.; Da Fonseca da Silva, L.; Medina-Castro, M.; Grayson, M.; Morell, V.; Palmer, A.; Sharma, M.; Stebler, J.; et al. A High-Fidelity Percutaneous Model Used to Demonstrate ECMO Cannulation. *The Journal of ExtraCorporeal Technology* **2021**, *53*, 208-213, doi:10.1182/ject-2100009.
11. Califia Patient Simulator. Available online: https://www.biomedsimulation.com/califia-3-0/ (accessed on 11 October 2022).
12. Califia Lung Simulator. Available online: https://www.biomedsimulation.com/califia-lung/ (accessed on 11 October 2022).
13. Alhomsi, Y.; Alsalemi, A.; Noorizadeh, M.; Bensaali, F.; Meskin, N.; Hssain, A.A. A Modular Approach for a Patient Unit for Extracorporeal Membrane Oxygenation Simulator. *Membranes* **2021**, *11*, 424, doi:10.3390/membranes11060424.
14. Parallel Simulator. Available online: https://www.chalicemedical.com/products/parallel-simulator/ (accessed on 12 October 2022).
15. Colasanti, S.; Piemonte, V.; Devolder, E.; Zieliński, K.; Vandendriessche, K.; Meyns, B.; Fresiello, L. Development of a computational simulator of the extracorporeal membrane oxygenation and its validation with in vitro measurements. *Artificial Organs* **2021**, *45*, 399-410, doi:10.1111/aor.13842.
16. ECMO Training Simulator. Available online: https://healtheng.illinois.edu/about/researchareas/simulationeducation/ecmo (accessed on 24 October 2022).
17. Lansdowne, W.; Machin, D.; Grant, D.J. Development of the Orpheus Perfusion Simulator for Use in High-fidelity Extracorporeal Membrane Oxygenation Simulation. *The Journal of ExtraCorporeal Technology* **2012**, *44*, 250-255.
18. Hybrids Vita. Available online: https://hybrids-vita.com/en.html (accessed on 30 November 2022).
19. MSE ECMO simulator. Available online: http://www.ecmosimulation.com/ (accessed on 17 December 2022).
20. Harvi ECMO. Available online: https://harvi.online/site/welcome/harvi-ecmo/ (accessed on 12 October 2022).

21. Zanella, A.; Salerno, D.; Scaravilli, V.; Giani, M.; Castagna, L.; Magni, F.; Carlesso, E.; Cadringher, P.; Bombino, M.; Grasselli, G.; et al. A mathematical model of oxygenation during venovenous extracorporeal membrane oxygenation support. *J Crit Care* **2016**, *36*, 178-186, doi:10.1016/j.jcrc.2016.07.008.
22. Allan, C.K.; Pigula, F.; Bacha, E.A.; Emani, S.; Fynn-Thompson, F.; Thiagarajan, R.R.; Imprescia, A.; Hayes, G.; Weinstock, P. An extracorporeal membrane oxygenation cannulation curriculum featuring a novel integrated skills trainer leads to improved performance among pediatric cardiac surgery trainees. *Simul Healthc* **2013**, *8*, 221-228, doi:10.1097/SIH.0b013e31828b4179.
23. RediStik ECMO Cannulation Trainer. Available online: https://www.youtube.com/watch?v=0wt5EHxH3CM (accessed on 11 November 2022).
24. Eigenflow ECMO Simulator. Available online: https://curtisliferesearch.com/eigenflow-ecmo-simulator/ (accessed on 10 October 2022).
25. ECMO Trainer Professional MK2. Available online: https://erler-zimmer.de/shop/en/10953 (accessed on 31 October 2022).
26. E-SIM Pro. Available online: https://www.thesimulatorcompany.com/e-sim-pro (accessed on 12 October 2022).
