# Supplementary material for: Simulators and Simulations for Extracorporeal Membrane Oxygenation: An ECMO Scoping Review
Source: J Clin Med. 2023 Feb 22;12(5):1765. doi: 10.3390/jcm12051765 (PMC10003420; doi:10.3390/jcm12051765)
Supplement: Supplementary file 1 [file jcm-12-01765-s001.zip › Table S1 Desk Research.pdf]

1. Materials and Methods

Following the PRISMA (Preferred Reporting Items for Systematic Reviews and Meta-Analyses) scoping guidelines [1], this scoping review aims to identify characteristics and specific training media to include in ECMO simulations and simulators (ECMO sims). In addition, desk research was carried out to gather relevant literature for recent developments [2-13] and commercially available ECMO sims [14-27].

1.1. Desk Research

The in- and exclusion criteria for the literature collection were as follows:

- Research material is derived from (online) databases, including: PubMed, Web of Science Core Collection, and Google (Scholar);
- Research material is taken from its original source;
- The literature contains information regarding ECMO sims;
- The information applies to the comparison of ECMO sims;
- The literature has not been retracted;
- No case studies are used unless the importance of the study can be argued;
- No review studies are included in the literature to process specific device developments;
- The language of the literature material should be available in English.

A list of key concepts related to ECMO sims was created, and for each key concept synonyms, narrow terms, and broader terms were drawn up and used in search queries (Table 1).

Table S1. List of key concepts with accompanying synonyms, narrower terms and broader terms used to make search queries in (online) databases, such as PubMed, Web of Science Core Collection, and Google (Scholar).

Key Concepts	Synonyms	Narrower Terms	Broader terms
Simulation	Model	Computation	Artificial
	Modeling	Computational	Copy
	Simulator	Computer-simulation	Imitation
		Numerical simulation	Mock-up
		Physical simulation	Resemblance
		Mannikin	Substitute
Physical	Material	Artificial blood	Authentic
	Substantial	Artificial tissue	Real
	Tangible		Un-imaginary
ECMO	Air support	Acute life support	ICU
	Artificial lung	Gas exchange	Hospital
	Breath support	Jetted outflow	Ventilator
	ECLS	Lungs	
	Oxygenator	Non-physiological flow	
	Respiratory support		

Lung	Pneumonic system<br>Pulmonary system<br>Pulmonic system<br>Respiratory system	Alveoli<br>Bronchial tubes<br>Bronchioles<br>Bronchus<br>Capillary (network)<br>Pulmonary artery<br>Pulmonary vein<br>Trachea	Air<br>Breathing<br>Organ<br>Oxygen
Device	Apparatus<br>Gadget<br>Instrument<br>Machine<br>Mechanism<br>Tool	Mechanics<br>Software<br>System	Innovation<br>Invention<br>Mechanics<br>Tactic<br>Technology
Production	Construction<br>Fabrication<br>Manufacture	Assembly<br>Attach<br>Constrain<br>Fixate<br>Machinery<br>Mould	Creation<br>Design<br>Establish<br>Generate<br>Invent<br>Organize

The search results' relevance was noted in a table to keep an overview of the processed work. E.g. the search query TITLE-ABS-KEY(ecmo AND Simulator) in Scopus Elsevier was sorted by relevance and gave 38 results of which 10 were relevant. The bibliography of all the relevant literature was read thoroughly to identify all the applicable literature. Selected literature was then reviewed by all authors and relevant information was summarized in the literature database. Examination of this literature lead to an overview of the developed and commercially available ECMO sims.

1.2. Fidelity classification

To distinguish the level of fidelity between the ECMO sims, fidelity was categorized into: low-fidelity, mid-fidelity, and high-fidelity. Furthermore, the overall fidelity was derived from the median of the definition-based fidelity, component fidelity, and customization fidelity.

1.2.1. Definition-based fidelity

To determine the fidelity classification of ECMO sims, existing definitions were used when possible, i.e. definition-based fidelity (DBF). These definitions have been derived from established dictionaries, standards, and literature, such as the oxford dictionary, the International Organization for Standardization (ISO), and The ELSO Red Book [28] (Table A1). According to the Healthcare Simulation Dictionary [29], DBF can be divided into four sub-categories: conceptual fidelity, functional fidelity, physical fidelity, and psychological fidelity. For conceptual fidelity, differentiation between computational, physical, or a combination of both was determined. This was deduced from the available information about the ECMO sim. The level of functional, physical, and psychological fidelity was assigned by the authors educated guess based on the available ECMO information. When the level of fidelity was determined to be low, a minus (-) was assigned. When mid, a plus-minus (+/-) was appointed, and a plus (+) was set to the ECMO sim when high. Eventually, the median of these last three fidelities was calculated to determine the DBF classification and agreed upon with all co-authors. As DBF classification is subjective, this classification

was weighed alongside the component fidelity and the customization fidelity for improved objectiveness.

1.2.2. Component fidelity

Component fidelity is based on the main components of ECMO: diagnostics, circuit priming, circuit monitoring, cannulation, connection ECMO/oxygenator, gas exchange, hemodynamics, weaning, decannulation, and (clinical) scenarios [30]. Based on the available information about the ECMO sim, the authors determined whether a component was included in the design or not. In case a component was included, it was specified if it was included computationally (C), physically (P), or as a combination of both. A total score out of 10 possible components determined whether the component fidelity is low, mid, or high. Low-fidelity was assigned to ECMO sims when the total ECMO components were ≤ 3, mid-fidelity was assigned when the total ECMO components was 4 to 7, while high fidelity was assigned when the total amount of ECMO components were ≥ 8.

1.2.3. Customization fidelity

Customization fidelity was based on the ability to adjust ECMO sim parameters to create more diverse patient-related scenarios. Similar to the component fidelity, the authors, based on the available ECMO sim information, determined whether a parameter was included in the design. The six parameters considered for ECMO sims were: sex, age, body size, race, disease and/or anatomy, and BMI / fat percentage. These parameters were chosen to influence the ECMO procedure. Race was left out of a secondary classification for computational or a combination of computational and physical ECMO sims without representation of the patient, resulting in five parameters. Low customization fidelity was based on ≤ 2/6 or 1/5, mid-fidelity customization was between 2/6 and 5/6 or between 1/5 and 5/5, while high fidelity customization was achieved when ≥ 5/6 or 5/5.

1.2.4. Overall fidelity

Finally, the median of the outcomes of all these fidelity types was calculated for each ECMO sim to determine the overall fidelity. Based on these outcomes, the ECMO sims were compared to each other and allocated to overall low-, mid-, and high-fidelity ECMO sims.

Appendix A

Established nomenclature based on existing literature (Table A1, left column) and slight changes based on our expert opinion when these definitions are applied to the field of ECMO simulation

Table A1. Nomenclature created out of existing definitions. The first column shows the word, second column cites the definition found in literature, the third column displays the source in which the definition was found, and the fourth column presents an altered definition in case the literature definitions did not correlate with the specific application of ECMO.

Word	Literature definition	Source	Our definition
Cannulation	"A thin tube inserted into a vein or body cavity to administer medication, drain off fluid, or insert a surgical instrument".	[31]	Technique to insert a tube, also known as cannulae, into a vessel (vein or artery). Cannulae are integral parts of the circuit and provide the intravascular connections to the patient.
	"Cannulae are integral parts of the circuit and provide the intravascular connections to the patient".	[30]	
(Clinical) Scenarios	"A deliberately designed simulation experience (also known as a case), that provides participants with an opportunity to meet identified objectives. The scenario provides a context for the simulation and can vary in length and complexity, depending on the objectives." [32-36]	Taken from source(s) in [37]	A deliberately designed simulation experience (also known as a case), that provides participants with an opportunity to meet identified objectives. The scenario provides a clinically plausible context for the simulation and can vary in length and complexity, depending on the objectives.
Circuit priming	"The prime is recirculated through a reservoir until all bubbles are removed". "The ECLS circuit is primed under sterile conditions".	[28]	The prime is recirculated through a reservoir until all bubbles are removed (de-airing) before connection to the patient". "The ECLS circuit is primed under sterile conditions"
Circuit monitoring	"a device used for observing, checking, or keeping a continuous record of something".	[31]	A method used for observing, checking, or keeping a continuous record of flows, pressures, blood features and other attributes.
	"Circuit monitoring is required to ensure the proper functionality of the circuit and to guide proper management. Common circuit monitoring techniques include flow, pressure, and saturation monitoring through integrated or external sensors".	[30]	Circuit monitoring is required to ensure the proper functionality of the circuit and to guide effective patient management. Common circuit monitoring techniques include integrated or external sensors to detect blood flow, pressures, pump speed (rotations per minute), and oxygen saturation.
Component fidelity	N/A	N/A	Fidelity based on the main components of ECMO: diagnostics, circuit priming, circuit monitoring, cannulation, connection ECMO/oxygenator, gas exchange, hemodynamics, weaning, decannulation, and (clinical) scenarios [42]

Computational (simulator)	"using or relating to computers" "relating to the process of mathematical calculation"	[31]	A simulation relating to the process of mathematical calculations performed by a computer.
Conceptual fidelity	"In health care simulation, ensures that all elements of the scenario relate to each other in a realistic way so that the case makes sense as a whole to the learner(s) (For example: Vital signs are consistent with the diagnosis)".	[29]	N/A
Connection oxygenator	"the action of linking one thing with another", in this case the oxygenator with the accompanying equipment.	[31]	N/A
Customization (fidelity)	"modify (something) to suit a particular individual or task"	[31]	"modify (something) to suit a particular individual or task", in this case the patient simulator related parameters: sex, age, size, race, disease and/or anatomy, and fat percentage/BMI.
Decannulation	"Extubation of a cannula"	[38]	Removal of a cannula.
Diagnosis/diagnostics	"The identification of the nature of an illness or other problem by examination of the symptoms".	[31]	N/A
Environmental fidelity	"The degree to which the simulated environment (manikin, room, tools, equipment, moulage, and sensory props) replicates reality and appearance of the real environment".	[29]	N/A
(Definition-based) Fidelity	"The ability of the simulation to reproduce the reactions, interactions, and responses of the real-world counterpart. It is not constrained to a certain type of simulation modality, and higher levels of fidelity are not required for a simulation to be successful".	[29]	N/A
Functional fidelity	"The degree in which the equipment used in the simulation responds to the participant's actions; e.g., a static ventilator would offer low functional fidelity compared to a working ventilator in a simulation requiring a ventilator alarm".	[29]	N/A
Gas exchange	"The ability to simulate lung function through the use of an artificial lung".	[30]	N/A
	"Oxygen and CO_2 diffuse between the gas and the blood as a function of the gradient between the partial pressures on each side".	[28]	
Hemodynamics	"Relating to the flow of blood within the organs and tissues of the body"	[31]	Basic measures of cardiovascular function, relating to blood flow and pressure in the vascular system, and perfusion within the organs and tissues of the body. The main factors involved are the pulsatile driving pressure generated by the heart, the flow characteristics of blood, and the geometric structure and mechanical properties of the vessels.
	"…the distribution of blood flow and blood pressure in the vascular system. The main factors involved are the pulsatile driving pressure	[39]	

	generated by the heart, the flow characteristics of blood, and the geometric structure and mechanical properties of the vessels. … In medical contexts, the term "hemodynamics" often refers to basic measures of cardiovascular function, such as arterial pressure or cardiac output."		
High-fidelity simulator	"A term often used to refer to the broad range of full-body manikins that have the ability to mimic, at a very high level, human body functions".	[29]	A term often used to refer to the broad range of full-body models or manikins that have the ability to mimic, at a very high level, human body functions.
High-fidelity simulation	"In health care simulation, high-fidelity refers to simulation experiences that are extremely realistic and provide a high level of interactivity and realism for the learner [40]. It can apply to any mode or method of simulation; for example: human, manikin, task trainer, or virtual reality."	Taken from source in [29]	N/A
Immersive simulation	"A simulation session influenced by participants' characteristics, experiences, level of training, and preparation for the case or task,. The perceived physical, conceptual and emotional fidelity, the appropriate level of challenge, and the simulators and actors can all affect the simulation experience" [41]	Taken from source in [29]	N/A
Low-fidelity	"Not needing to be controlled or programmed externally for the learner to participate [42]; examples include case studies, role playing, or task trainers used to support students or professionals in learning a clinical situation or practice (Adapted from [43])" .	Taken from source in [29]	N/A
Manikin /mannikin	"A jointed model of the human body, used in anatomy or as an artist's lay figure".	[31]	N/A
Mid-fidelity	"Use of computer technology to assist learners in developing competencies in skills"	[44]	Use of (computer) technology to assist learners in developing competencies in skills.
Overall fidelity	N/A	N/A	The definite fidelity of an ECMO simulation or simulator derived from definition-based, component, and customization fidelity.
Physical fidelity	"The degree to which the simulation looks, sounds, and feels like the actual task" [45]	Taken from source in [29]	N/A
Physical (simulator)	See also Physical fidelity.	N/A	Simulations substituting an object (or person) for an, often simplified, material representation as a real-life counterpart.

Psychological fidelity	"The degree of perceived realism, including psychological factors such as emotions, beliefs, and self-awareness of participants in simulation scenarios" [46].	Taken from source in [29]	N/A
Realism	"Refers to the physical characteristics of the activity, semantical aspects of the activity (theories and conceptual relations – if A happens then B occurs), and/or the phenomenal aspects of the activity (emotions, beliefs, and thoughts experienced)."	[29]	N/A
Simulation	"A technique that creates a situation or environment to allow persons to experience a representation of a real event for the purpose of practice, learning, evaluation, testing, or to gain understanding of systems or human actions".	[29]	N/A
Simulation fidelity	"The physical, semantic, emotional, and experiential accuracy that allows persons to experience a simulation as if they were operating in an actual activity (SSH)"	[29]	N/A
Weaning	"Accustom (someone) to managing without something which they have become dependent on"<br>"The transition to normal patient circulation and physiology from extracorporeal circulation".	[31]<br>[47]	Accustom (someone) to managing without ECMO which they have become dependent on. Also known as: the transition to normal patient circulation and physiology from extracorporeal circulation.

References

1. Tricco, A.C.; Lillie, E.; Zarin, W.; O'Brien, K.K.; Colquhoun, H.; Levac, D.; Moher, D.; Peters, M.D.J.; Horsley, T.; Weeks, L.; et al. PRISMA Extension for Scoping Reviews (PRISMA-ScR): Checklist and Explanation. *Ann Intern Med* **2018**, *169*, 467-473, doi:10.7326/M18-0850.
2. Allan, C.K.; Pigula, F.; Bacha, E.A.; Emani, S.; Fynn-Thompson, F.; Thiagarajan, R.R.; Imprescia, A.; Hayes, G.; Weinstock, P. An extracorporeal membrane oxygenation cannulation curriculum featuring a novel integrated skills trainer leads to improved performance among pediatric cardiac surgery trainees. *Simul Healthc* **2013**, *8*, 221-228, doi:10.1097/SIH.0b013e31828b4179.
3. Alhomsi, Y.; Alsalemi, A.; Noorizadeh, M.; Bensaali, F.; Meskin, N.; Hssain, A.A. A Modular Approach for a Patient Unit for Extracorporeal Membrane Oxygenation Simulator. *Membranes* **2021**, *11*, 424, doi:10.3390/membranes11060424.
4. Broman, M.; Frenckner, B.; Bjällmark, A.; Broomé, M. Recirculation during Veno-Venous Extra-Corporeal Membrane Oxygenation – a Simulation Study. *The International Journal of Artificial Organs* **2015**, *38*, 23-30, doi:10.5301/ijao.5000373.
5. Colasanti, S.; Piemonte, V.; Devolder, E.; Zieliński, K.; Vandendriessche, K.; Meyns, B.; Fresiello, L. Development of a computational simulator of the extracorporeal membrane oxygenation and its validation with in vitro measurements. *Artificial Organs* **2021**, *45*, 399-410, doi:10.1111/aor.13842.
6. Endo, T.; Kagaya, Y.; Arata, Y.; Imai, H. Long-term efficacy of an extracorporeal membrane oxygenation simulation with a novel, low-cost vascular model "Endo-Circuit". *Acute Medicine & Surgery* **2017**, *4*, 79-88, doi:10.1002/ams2.236.
7. Lansdowne, W.; Machin, D.; Grant, D.J. Development of the Orpheus Perfusion Simulator for Use in High-fidelity Extracorporeal Membrane Oxygenation Simulation. *The Journal of ExtraCorporeal Technology* **2012**, *44*, 250-255.
8. Mahmoud, A.; Hssain, A.A.; Alinier, G.; Hassan, I.; Khurshid, U.; Abducarim, A.; Mahmud, S.; Abdallah, O.; Mohamed, E.; Alsalemi, A.; et al. Preliminary Implementation of the Next Generation Cannulation Simulator. 2019, 2019.
9. Palmer, D.; Aspenleiter, M.; da Silva, J.; Castro-Medina, M.; Morell, V.; Sharma, M.; Viegas, M. A High-Fidelity Surgical Model and Perfusion Simulator Used to Demonstrate ECMO Cannulation, Initiation, and Stabilization. *The Journal of ExtraCorporeal Technology* **2019**, *51*, 6.
10. Palmer, D.; Aspenleiter, M.; da Silva, J.; Da Fonseca da Silva, L.; Medina-Castro, M.; Grayson, M.; Morell, V.; Palmer, A.; Sharma, M.; Stebler, J.; et al. A High-Fidelity Percutaneous Model Used to Demonstrate ECMO Cannulation. *The Journal of ExtraCorporeal Technology* **2021**, *53*, 208-213, doi:10.1182/ject-2100009.
11. Puslecki, M.; Ligowski, M.; Kiel, M.; Dabrowski, M.; Stefaniak, S.; Sip, M.; Maciejewski, A.; Dabrowska, A.; Kiel-Puslecka, I.; Kłosiewicz, T.; et al. Prototype of extracorporeal membrane oxygenation (ECMO) therapy simulator used in regional ECMO program. *Journal of Thoracic Disease* **2018**, *10*, 5073-5079, doi:10.21037/jtd.2018.07.25.
12. Thompson, J.L.; Grisham, L.M.; Scott, J.; Mogan, C.; Prescher, H.; Biffar, D.; Jarred, J.; Meyer, R.J.; Hamilton, A.J. Construction of a reusable, high-fidelity model to enhance extracorporeal membrane oxygenation training through simulation. *Adv Neonatal Care* **2014**, *14*, 103-109, doi:10.1097/ANC.0000000000000054.
13. Zanella, A.; Salerno, D.; Scaravilli, V.; Giani, M.; Castagna, L.; Magni, F.; Carlesso, E.; Cadringher, P.; Bombino, M.; Grasselli, G.; et al. A mathematical model of oxygenation during venovenous extracorporeal membrane oxygenation support. *J Crit Care* **2016**, *36*, 178-186, doi:10.1016/j.jcrc.2016.07.008.
14. 3-Dmed. ECMO Simulation Kit. Available online: https://www.3-dmed.com/wp-content/uploads/2019/05/ECMO-Simulation-Kit.pdf (accessed on 10 October 2022).
15. Califia Patient Simulator. Available online: https://www.biomedsimulation.com/califia-3-0/ (accessed on 11 October 2022).
16. Califia Lung Simulator. Available online: https://www.biomedsimulation.com/califia-lung/ (accessed on 11 October 2022).

17. Parallel Simulator. Available online: https://www.chalicemedical.com/products/parallel-simulator/ (accessed on 12 October 2022).
18. ECMO Trainer Evolution III. Available online: https://www.crea-plast.com/en/45-ecmo-trainer (accessed on 12 October 2022).
19. Eigenflow ECMO Simulator. Available online: https://curtisliferesearch.com/eigenflow-ecmo-simulator/ (accessed on 10 October 2022).
20. ECMO Trainer Professionl MK2. Available online: https://erler-zimmer.de/shop/en/10953 (accessed on 31 October 2022).
21. ECMO Training Simulator. Available online: https://healtheng.illinois.edu/about/researchareas/simulationeducation/ecmo (accessed on 24 October 2022).
22. Hybrids Vita. Available online: https://hybrids-vita.com/en.html (accessed on 30 November 2022).
23. MSE ECMO simulator. Available online: http://www.ecmosimulation.com/ (accessed on 17 December 2022).
24. Harvi ECMO. Available online: https://harvi.online/site/welcome/harvi-ecmo/ (accessed on 12 October 2022).
25. ECMOjo Simulator. Available online: https://ecmojo.sourceforge.net/ (accessed on 12 October 2022).
26. RediStik ECMO Cannulation Trainer. Available online: https://www.youtube.com/watch?v=0wt5EHxH3CM (accessed on 11 November 2022).
27. E-SIM Pro. Available online: https://www.thesimulatorcompany.com/e-sim-pro (accessed on 12 October 2022).
28. Brogan, T.V.; Lequier, L.; Lorusso, R.; MacLaren, G.; Peek, G. *Extracorporeal Life Support: The ELSO Red Book*, 5th ed.; USA, 2017.
29. Lioce, L.; Downing, D.; Chang, T.P.; Robertson, J.M.; Anderson, M.; Diaz, D.A.; Spain, A.E.; (2020), T.T.a.C.W.G. *Healthcare Simulation Dictionary*, 2nd ed.; Lioce, L., Lopreiato, J., Spain, A.E., Eds.; MD: Agency for Healthcare Research and Quality: Rockville, 2020.
30. Vercaemst, L.; Maul, T.M.; Arens, J.; Toomasian, J.M. Chapter 3: The Circuit. In *Extracorporeal Life Support: The ELSO Red Book*, 6th ed.; 2022.
31. Oxford dictionary of English. **2022**.
32. Waxman, K.T. The development of evidence-based clinical simulation scenarios: guidelines for nurse educators. *J Nurs Educ* **2010**, *49*, 29-35, doi:10.3928/01484834-20090916-07.
33. Rudolph, J.W.; Raemer, D.B.; Simon, R. Establishing a safe container for learning in simulation: the role of the presimulation briefing. *Simul Healthc* **2014**, *9*, 339-349, doi:10.1097/SIH.0000000000000047.
34. Alinier, G. Developing High-Fidelity Health Care Simulation Scenarios: A Guide for Educators and Professionals. *Simulation & Gaming* **2010**, *42*, 9-26, doi:10.1177/1046878109355683.
35. Aschenbrenner, D.S.; Milgrom, L.B.; Settles, J. Designing simulation scenarios to promote learning. In Proceedings of the Simulation in nursing education: From conceptualization to evaluation, New York, 2012.
36. Lioce, L.; Meakim, C.H.; Fey, M.K.; Chmil, J.V.; Mariani, B.; Alinier, G. Standards of Best Practice: Simulation Standard IX: Simulation Design. *Clinical Simulation in Nursing* **2015**, *11*, 309-315, doi:10.1016/j.ecns.2015.03.005.
37. INACSL Standards of Best Practice: Simulation Simulation Glossary. *Clinical Simulation in Nursing* **2016**, *12*, S39-S47, doi:10.1016/j.ecns.2016.09.012.
38. Miller-Keane; Marie T. O'Toole. *Miller-Keane Encyclopedia & Dictionary of Medicine, Nursing & Allied Health*, 7th ed.; Elsevier: 2003; p. 2344.
39. Secomb, T.W. Hemodynamics. *Compr Physiol* **2016**, *6*, 975-1003, doi:10.1002/cphy.c150038.
40. INACSL Standards of Best Practice: Simulation sm : Operations. *Clinical Simulation in Nursing* **2017**, *13*, 681-687, doi:10.1016/j.ecns.2017.10.005.

41. Hamstra, S.J.; Brydges, R.; Hatala, R.; Zendejas, B.; Cook, D.A. Reconsidering fidelity in simulation-based training. *Acad Med* **2014**, *89*, 387-392, doi:10.1097/ACM.0000000000000130.
42. Palaganas, J.C.; Maxworthy, J.C.; Epps, C.A.; Mancini, M.E. *Defining Excellence in Simulation Programs*, 1st ed.; LWW: 2014.
43. National League for Nursing Simulation Innovation Resource Center (NLN-SIRC). Available online: http://sirc.nln.org/mod/glossary/view.php?id¼183 (accessed on 8 November 2022).
44. Akhtar-Danesh, N.; Baxter, P.; Valaitis, R.K.; Stanyon, W.; Sproul, S. Nurse Faculty Perceptions of Simulation Use in Nursing Education. *Western J Nurs Res* **2009**, *31*, 312-329, doi:10.1177/0193945908328264.
45. Alexander, A.; Sidman, J.; Brunyé, T.T.; Weil, S.A. From Gaming to Training: A Review of Studies on Fidelity, Immersion, Presence, and Buyin and Their Effects on Transfer in PC-Based Simulations and Games. **2005**.
46. Dieckmann, P.; Rall, M. Designing a Scenario as a Simulated Clinical Experience: The TuPASS Scenario Script. *Clinical Simulation Operations, Engineering and Management* **2008**, 541-550, doi:10.1016/B978-012372531-8.50096-0.
47. ISO. ISO/TS 23810:2018 Cardiovascular implants and artificial organs — Checklists for use of extracorporeal circulation equipment. **2018**.
