# Supplementary material for: Simulators and Simulations for Extracorporeal Membrane Oxygenation: An ECMO Scoping Review
Source: J Clin Med. 2023 Feb 22;12(5):1765. doi: 10.3390/jcm12051765 (PMC10003420; doi:10.3390/jcm12051765)
Supplement: Supplementary file 1 [file jcm-12-01765-s001.zip › Table S3 Component Fidelity.pdf]

Table S3. Component fidelity (right column) is established by counting the total ECMO process components (diagnostics, circuit priming, circuit monitoring, cannulation, gas exchange, hemodynamics, weaning, decannulation, ad (clinical) scenarios). The components are included computationally (C), physically (P), or computationally and physically (C+P). A total score out of these 10 possible components determined the classification is low-, mid-, or high-fidelity. Low-fidelity was assigned to ECMO sims when the total ECMO components were ≤ 3, mid-fidelity was assigned when the total ECMO components was 4 to 7, while high fidelity was assigned when the total amount of ECMO components were ≥ 8.

Source	Device	Diagnostics (C, P, C+P)	Circuit priming (C, P, C+P)	Circuit monitoring (C, P, C+P)	Cannulation (C, P, C+P)	Connection ECMO / oxygenator (C, P, C+P)	Gas exchange (C, P, C+P)	Hemodynamics (C, P, C+P)	Weaning (C, P, C+P)	Decannulation (C, P, C+P)	(Clinical) scenarios (C, P, C+P)	Total ECMO process components	Component fidelity (Low-Mid-High)
3-Dmed [1]	ECMO Simulation Kit	N/A	N/A	N/A	P	N/A	N/A	N/A	N/A	N/A	N/A	1/10	Low
Allan, *et al.* [2]	Integrated Skills Trainer	N/A	N/A	N/A	P	P	N/A	P	N/A	N/A	N/A	3/10	Low
Alhomsi, *et al.* [3]	Modular ECMO simulator	C+P	N/A	P	N/A	P	P	P	N/A	N/A	C+P	6/10	Mid
BioMed Simulations [4]	Califia Patient Simulator	C	N/A	P	N/A	P	N/A	C	N/A	N/A	C	5/10	Mid
BioMed Simulations [5]	Califia Lung Simulator	C	N/A	P	N/A	P	C	C	N/A	N/A	C	6/10	Mid
Broman, *et al.* [6]	Aplysia	N/A	N/A	C	C	N/A	C	C	N/A	N/A	C	5/10	Mid
Chalice [7]	Parallel Simulator	C	N/A	C+P	N/A	P	C	C	N/A	N/A	C	6/10	Mid
Colasanti, *et al.* [8]	Computational ECMO Simulator	C	N/A	C	N/A	N/A	N/A	C	N/A	N/A	C	4/10	Mid
Creaplast [9]	ECMO Trainer Evolution III	N/A	N/A	N/A	P	N/A	N/A	P	N/A	N/A	P	3/10	Low
Curtis Life Research [10]	Eigenflow 2 ADVANCED	C	N/A	C	P	N/A	C	C	N/A	N/A	C+P	6/10	Mid
Endo, *et al.* [11]	Endo Circuit	N/A	N/A	N/A	P	N/A	N/A	N/A	N/A	N/A	N/A	1/10	Low
Erler Zimmer [12]	ECMO Trainer Professional MK2	N/A	N/A	N/A	P	P	N/A	P	N/A	P	C+P	6/10	Mid
Health Care Engineering Systems Center [13]	ECMO Training Simulator	N/A	N/A	N/A	P	N/A	P	P	N/A	N/A	C+P	4/10	Mid
Lansdowne, *et al.* [14]	Orpheus perfusion simulator	C+P	N/A	C+P	N/A	P	N/A	C+P	N/A	N/A	C+P	5/10	Mid

Mahmoud, *et al.* [15]	Cannulation simulator	N/A	N/A	N/A	C+P	P	N/A	N/A	N/A	N/A	C+P	3/10	Low
Medical Simulator [16]	Hybrids Vita	N/A	N/A	C+P	C	P	C+P	C+P	N/A	N/A	C+P	6/10	Mid
MSE [17]	Adult ECMO Simulator	C	N/A	C	N/A	N/A	C	C	N/A	N/A	C	5/10	Mid
Palmer, *et al.* [18]	Surgical model	N/A	N/A	N/A	P	P	N/A	N/A	N/A	N/A	N/A	2/10	Low
Palmer, *et al.* [19]	Percutaneous model	N/A	N/A	N/A	P	P	N/A	N/A	N/A	N/A	N/A	2/10	Low
Puslecki, *et al.* [20]	ECMO therapy simulator	C+P	N/A	P	P	P	N/A	C	N/A	C+P	C+P	7/10	Mid
PVLoops [21]	Harvi ECMO	C	N/A	C	C	N/A	C	C	N/A	N/A	C	6/10	Mid
Telehealth Research Institute [22]	ECMOjo	C	N/A	C	N/A	N/A	C	C	N/A	N/A	C	6/10	Mid
Texas Children's Hospital [23]	RediStick ECMO Cannulation Trainer	N/A	P	N/A	P	P	N/A	N/A	N/A	N/A	N/A	3/10	Low
The Simulator Company [24]	E-Sim Pro	N/A	N/A	N/A	P	P	N/A	C	N/A	N/A	C	4/10	Mid
Thompson, *et al.* [25]	ECMO Initiation Simulator	N/A	N/A	N/A	P	P	N/A	N/A	N/A	C	N/A	2/10	Low
Zanella, *et al.* [26]	Mathematical ECMO model	C	N/A	C	N/A	N/A	C	C	N/A	N/A	C	5/10	Mid

References

1. 3-Dmed. ECMO Simulation Kit. Available online: (accessed on 10 October 2022).
2. Allan, C.K.; Pigula, F.; Bacha, E.A.; Emani, S.; Fynn-Thompson, F.; Thiagarajan, R.R.; Imprescia, A.; Hayes, G.; Weinstock, P. An extracorporeal membrane oxygenation cannulation curriculum featuring a novel integrated skills trainer leads to improved performance among pediatric cardiac surgery trainees. *Simul Healthc* **2013**, *8*, 221-228, doi:10.1097/SIH.0b013e31828b4179.
3. Alhomsi, Y.; Alsalemi, A.; Noorizadeh, M.; Bensaali, F.; Meskin, N.; Hssain, A.A. A Modular Approach for a Patient Unit for Extracorporeal Membrane Oxygenation Simulator. *Membranes* **2021**, *11*, 424, doi:10.3390/membranes11060424.
4. Califia Patient Simulator. (accessed on 11 October 2022).
5. Califia Lung Simulator. Available online: (accessed on 11 October 2022).
6. Broman, M.; Frenckner, B.; Bjällmark, A.; Broomé, M. Recirculation during Veno-Venous Extra-Corporeal Membrane Oxygenation – a Simulation Study. *The International Journal of Artificial Organs* **2015**, *38*, 23-30, doi:10.5301/ijao.5000373.
7. Parallel Simulator. (accessed on 12 October 2022).
8. Colasanti, S.; Piemonte, V.; Devolder, E.; Zieliński, K.; Vandendriessche, K.; Meyns, B.; Fresiello, L. Development of a computational simulator of the extracorporeal membrane oxygenation and its validation with in vitro measurements. *Artificial Organs* **2021**, *45*, 399-410, doi:10.1111/aor.13842.
9. ECMO Trainer Evolution III. (accessed on 12 October 2022).
10. Eigenflow ECMO Simulator. Available online: (accessed on 10 October 2022).
11. Endo, T.; Kagaya, Y.; Arata, Y.; Imai, H. Long-term efficacy of an extracorporeal membrane oxygenation simulation with a novel, low-cost vascular model "Endo-Circuit". *Acute Medicine & Surgery* **2017**, *4*, 79-88, doi:10.1002/ams2.236.
12. ECMO Trainer Professionl MK2. (accessed on 31 October 2022).
13. ECMO Training Simulator. Available online: (accessed on 24 October 2022).
14. Lansdowne, W.; Machin, D.; Grant, D.J. Development of the Orpheus Perfusion Simulator for Use in High-fidelity Extracorporeal Membrane Oxygenation Simulation. *The Journal of ExtraCorporeal Technology* **2012**, *44*, 250-255.
15. Mahmoud, A.; Hssain, A.A.; Alinier, G.; Hassan, I.; Khurshid, U.; Abducarim, A.; Mahmud, S.; Abdallah, O.; Mohamed, E.; Alsalemi, A.; et al. Preliminary Implementation of the Next Generation Cannulation Simulator. 2019, 2019.
16. Hybrids Vita. (accessed on 30 November 2022).

17. MSE ECMO simulator. Available online: (accessed on 17 December 2022).
18. Palmer, D.; Aspenleiter, M.; da Silva, J.; Castro-Medina, M.; Morell, V.; Sharma, M.; Viegas, M. A High-Fidelity Surgical Model and Perfusion Simulator Used to Demonstrate ECMO Cannulation, Initiation, and Stabilization. *The Journal of ExtraCorporeal Technology* **2019**, *51*, 6.
19. Palmer, D.; Aspenleiter, M.; da Silva, J.; Da Fonseca da Silva, L.; Medina-Castro, M.; Grayson, M.; Morell, V.; Palmer, A.; Sharma, M.; Stebler, J.; et al. A High-Fidelity Percutaneous Model Used to Demonstrate ECMO Cannulation. *The Journal of ExtraCorporeal Technology* **2021**, *53*, 208-213, doi:10.1182/ject-2100009.
20. Puslecki, M.; Ligowski, M.; Kiel, M.; Dabrowski, M.; Stefaniak, S.; Sip, M.; Maciejewski, A.; Dabrowska, A.; Kiel-Puslecka, I.; Kłosiewicz, T.; et al. Prototype of extracorporeal membrane oxygenation (ECMO) therapy simulator used in regional ECMO program. *Journal of Thoracic Disease* **2018**, *10*, 5073-5079, doi:10.21037/jtd.2018.07.25.
21. Harvi ECMO. (accessed on 12 October 2022).
Available online: (accessed on 12 October 2022).
25. Thompson, J.L.; Grisham, L.M.; Scott, J.; Mogan, C.; Prescher, H.; Biffar, D.; Jarred, J.; Meyer, R.J.; Hamilton, A.J. Construction of a reusable, high-fidelity model to enhance extracorporeal membrane oxygenation training through simulation. *Adv Neonatal Care* **2014**, *14*, 103-109, doi:10.1097/ANC.0000000000000054.
26. Zanella, A.; Salerno, D.; Scaravilli, V.; Giani, M.; Castagna, L.; Magni, F.; Carlesso, E.; Cadringher, P.; Bombino, M.; Grasselli, G.; et al. A mathematical model of oxygenation during venovenous extracorporeal membrane oxygenation support. *J Crit Care* **2016**, *36*, 178-186, doi:10.1016/j.jcrc.2016.07.008.
