# Supplementary material for: Simulators and Simulations for Extracorporeal Membrane Oxygenation: An ECMO Scoping Review
Source: J Clin Med. 2023 Feb 22;12(5):1765. doi: 10.3390/jcm12051765 (PMC10003420; doi:10.3390/jcm12051765)
Supplement: Supplementary file 1 [file jcm-12-01765-s001.zip › Table S4 Customization Fidelity.pdf]

Table S4. Customization fidelity (right column) is established by counting the total customization parameters (sex, age, size, race, disease and/or anatomy, and BMI / fat percentage). The parameter race was left out of the classification for computational or a combination of computational and physical ECMO sims without representation of the patient. A total score out of 6 (or 5) determined the classification is low-, mid-, or high-fidelity. Low-fidelity was based on ≤ 2/6 or 1/5 , mid-fidelity was between 2/6 and 5/6 or between 1/5 and 5/5, while high fidelity customization was achieved when ≥ 5/6 or 5/5.

Source	Device	Visual representation of patient (Yes or No)	Computational and/or Physical Simulation (C, P, or C+P)	Sex (Yes or No)	Age (Yes or No)	Size (body height) (Yes or No)	Race (Yes or No)	Disease and/or anatomy (Yes or No)	BMI / Fat percentage (Yes or No)	Total customization parameters	Customization fidelity (Low-Mid-High)
3-Dmed [1]	ECMO Simulation Kit	Yes	P	No	No	No	No	No	No	0/6	Low
Allan, *et al.* [2]	Integrated Skills Trainer	Yes	P	No	No	No	No	No	No	0/6	Low
Alhomsi, *et al.* [3]	Modular ECMO simulator	Yes	C+P	No	No	No	No	No	No	0/6	Low
BioMed Simulations [4]	Califia Patient Simulator	No	C	No	Yes	Yes	N/A	Yes	No	3/5	Mid
BioMed Simulations [5]	Califia Lung Simulator	No	C	No	Yes	Yes	N/A	Yes	No	3/5	Mid
Broman, *et al.* [6]	Aplysia	No	C	No	No	No	N/A	No	No	0/5	Low
Chalice [7]	Parallel Simulator	No	C	No	No	Yes	N/A	Yes	No	2/5	Low
Colasanti, *et al.* [8]	Computational ECMO Simulator	No	C	No	No	No	N/A	Yes	No	1/5	Low
Creaplast [9]	ECMO Trainer Evolution III	Yes	C+P	No	No	No	No	No	No	0/6	Low
Curtis Life Research [10]	Eigenflow 2 ADVANCED	Yes	C+P	No	No	Yes	No	No	No	1/6	Low
Endo, *et al.* [11]	Endo Circuit	Yes	P	No	No	No	No	No	No	0/6	Low
Erler Zimmer [12]	ECMO Trainer Professional MK2	Yes	C+P	No	No	No	No	No	No	0/6	Low
Health Care Engineering Systems Center [13]	ECMO Training Simulator	Yes	P	No	No	No	No	Yes	No	1/6	Low
Lansdowne, *et al.* [14]	Orpheus perfusion simulator	No	C	No	No	No	N/A	Yes	No	1/5	Low
Mahmoud, *et al.* [15]	Cannulation simulator	Yes	C+P	No	No	No	Yes	No	No	1/6	Low

Medical Simulator [16]	Hybrids Vita	No	C+P	No	No	Yes	N/A	No	No	1/5	Low
MSE [17]	Adult ECMO Simulator	No	C	No	No	No	N/A	Yes	No	1/5	Low
Palmer, *et al.* [18]	Surgical model	Yes	P	No	No	No	No	No	No	0/6	Low
Palmer, *et al.* [19]	Percutaneous model	Yes	P	No	No	No	No	No	No	0/6	Low
Puslecki, *et al.* [20]	ECMO therapy simulator	No	C+P	No	No	No	N/A	No	No	0/5	Low
PVLoops [21]	Harvi ECMO	Yes	C	No	No	No	No	Yes	Yes	2/6	Low
Telehealth Research Institute [22]	ECMOjo	Yes	C	No	No	No	No	No	No	0/6	Low
Texas Children's Hospital [23]	RediStick ECMO Cannulation Trainer	Yes	P	No	No	No	No	No	No	0/5	Low
The Simulator Company [24]	E-Sim Pro	Yes	C+P	No	No	No	No	Yes	No	1/6	Low
Thompson, *et al.* [25]	ECMO Initiation Simulator	Yes	P	No	No	No	No	No	No	0/6	Low
Zanella, *et al.* [26]	Mathematical ECMO model	Yes	C	No	Yes	Yes	No	Yes	No	3/6	Mid

References

1. 3-Dmed. ECMO Simulation Kit. Available online: https://www.3-dmed.com/wp-content/uploads/2019/05/ECMO-Simulation-Kit.pdf (accessed on 10 October 2022).
2. Allan, C.K.; Pigula, F.; Bacha, E.A.; Emani, S.; Fynn-Thompson, F.; Thiagarajan, R.R.; Imprescia, A.; Hayes, G.; Weinstock, P. An extracorporeal membrane oxygenation cannulation curriculum featuring a novel integrated skills trainer leads to improved performance among pediatric cardiac surgery trainees. *Simul Healthc* **2013**, *8*, 221-228, doi:10.1097/SIH.0b013e31828b4179.
3. Alhomsi, Y.; Alsalemi, A.; Noorizadeh, M.; Bensaali, F.; Meskin, N.; Hssain, A.A. A Modular Approach for a Patient Unit for Extracorporeal Membrane Oxygenation Simulator. *Membranes* **2021**, *11*, 424, doi:10.3390/membranes11060424.
4. Califia Patient Simulator. Available online: https://www.biomedsimulation.com/califia-3-0/ (accessed on 11 October 2022).
5. Califia Lung Simulator. Available online: https://www.biomedsimulation.com/califia-lung/ (accessed on 11 October 2022).
6. Broman, M.; Frenckner, B.; Bjällmark, A.; Broomé, M. Recirculation during Veno-Venous Extra-Corporeal Membrane Oxygenation – a Simulation Study. *The International Journal of Artificial Organs* **2015**, *38*, 23-30, doi:10.5301/ijao.5000373.
7. Parallel Simulator. Available online: https://www.chalicemedical.com/products/parallel-simulator/ (accessed on 12 October 2022).
8. Colasanti, S.; Piemonte, V.; Devolder, E.; Zieliński, K.; Vandendriessche, K.; Meyns, B.; Fresiello, L. Development of a computational simulator of the extracorporeal membrane oxygenation and its validation with in vitro measurements. *Artificial Organs* **2021**, *45*, 399-410, doi:10.1111/aor.13842.
9. ECMO Trainer Evolution III. Available online: https://www.crea-plast.com/en/45-ecmo-trainer (accessed on 12 October 2022).
10. Eigenflow ECMO Simulator. Available online: https://curtisliferesearch.com/eigenflow-ecmo-simulator/ (accessed on 10 October 2022).
11. Endo, T.; Kagaya, Y.; Arata, Y.; Imai, H. Long-term efficacy of an extracorporeal membrane oxygenation simulation with a novel, low-cost vascular model "Endo-Circuit". *Acute Medicine & Surgery* **2017**, *4*, 79-88, doi:10.1002/ams2.236.
12. ECMO Trainer Professionl MK2. Available online: https://erler-zimmer.de/shop/en/10953 (accessed on 31 October 2022).
13. ECMO Training Simulator. Available online: https://healtheng.illinois.edu/about/researchareas/simulationeducation/ecmo (accessed on 24 October 2022).
14. Lansdowne, W.; Machin, D.; Grant, D.J. Development of the Orpheus Perfusion Simulator for Use in High-fidelity Extracorporeal Membrane Oxygenation Simulation. *The Journal of ExtraCorporeal Technology* **2012**, *44*, 250-255.
15. Mahmoud, A.; Hssain, A.A.; Alinier, G.; Hassan, I.; Khurshid, U.; Abducarim, A.; Mahmud, S.; Abdallah, O.; Mohamed, E.; Alsalemi, A.; et al. Preliminary Implementation of the Next Generation Cannulation Simulator. 2019, 2019.
16. Hybrids Vita. Available online: https://hybrids-vita.com/en.html (accessed on 30 November 2022).

17. MSE ECMO simulator. Available online: http://www.ecmosimulation.com/ (accessed on 17 December 2022).
18. Palmer, D.; Aspenleiter, M.; da Silva, J.; Castro-Medina, M.; Morell, V.; Sharma, M.; Viegas, M. A High-Fidelity Surgical Model and Perfusion Simulator Used to Demonstrate ECMO Cannulation, Initiation, and Stabilization. *The Journal of ExtraCorporeal Technology* **2019**, *51*, 6.
19. Palmer, D.; Aspenleiter, M.; da Silva, J.; Da Fonseca da Silva, L.; Medina-Castro, M.; Grayson, M.; Morell, V.; Palmer, A.; Sharma, M.; Stebler, J.; et al. A High-Fidelity Percutaneous Model Used to Demonstrate ECMO Cannulation. *The Journal of ExtraCorporeal Technology* **2021**, *53*, 208-213, doi:10.1182/ject-2100009.
20. Puslecki, M.; Ligowski, M.; Kiel, M.; Dabrowski, M.; Stefaniak, S.; Sip, M.; Maciejewski, A.; Dabrowska, A.; Kiel-Puslecka, I.; Kłosiewicz, T.; et al. Prototype of extracorporeal membrane oxygenation (ECMO) therapy simulator used in regional ECMO program. *Journal of Thoracic Disease* **2018**, *10*, 5073-5079, doi:10.21037/jtd.2018.07.25.
21. Harvi ECMO. Available online: https://harvi.online/site/welcome/harvi-ecmo/ (accessed on 12 October 2022).
22. ECMOjo Simulator. Available online: https://ecmojo.sourceforge.net/ (accessed on 12 October 2022).
23. RediStik ECMO Cannulation Trainer. Available online: https://www.youtube.com/watch?v=0wt5EHxH3CM (accessed on 11 November 2022).
24. E-SIM Pro. Available online: https://www.thesimulatorcompany.com/e-sim-pro (accessed on 12 October 2022).
25. Thompson, J.L.; Grisham, L.M.; Scott, J.; Mogan, C.; Prescher, H.; Biffar, D.; Jarred, J.; Meyer, R.J.; Hamilton, A.J. Construction of a reusable, high-fidelity model to enhance extracorporeal membrane oxygenation training through simulation. *Adv Neonatal Care* **2014**, *14*, 103-109, doi:10.1097/ANC.0000000000000054.
26. Zanella, A.; Salerno, D.; Scaravilli, V.; Giani, M.; Castagna, L.; Magni, F.; Carlesso, E.; Cadringher, P.; Bombino, M.; Grasselli, G.; et al. A mathematical model of oxygenation during venovenous extracorporeal membrane oxygenation support. *J Crit Care* **2016**, *36*, 178-186, doi:10.1016/j.jcrc.2016.07.008.
